# Comparative Analysis of Households Solid Waste Management in Rural and Urban Ghana

**DOI:** 10.1155/2016/5780258

**Published:** 2016-10-11

**Authors:** Simon Boateng, Prince Amoako, Divine Odame Appiah, Adjoa Afriyie Poku, Emmanuel Kofi Garsonu

**Affiliations:** ^1^Department of Geography and Rural Development, Kwame Nkrumah University of Science and Technology, Kumasi, Ghana; ^2^Department of Economics, Kwame Nkrumah University of Science and Technology, Kumasi, Ghana; ^3^Department of Geography Education, University of Education, Winneba, Ghana

## Abstract

The comparative analysis of solid waste management between rural and urban Ghana is largely lacking. This study investigated the solid waste situation and the organisation of solid waste management in both urban and rural settings from the perspective of households. The study employed cross-sectional survey covering both rural and urban districts in the Ashanti and Greater Accra Regions of Ghana. The study systematically sampled houses from which 400 households and respondents were randomly selected. Pearson's Chi square test was used to compare demographic and socioeconomic variables in rural and urban areas. Multivariate Test, Tests of Between-Subjects Effects, and Pair-Wise Comparisons were performed through one-way MANOVA to determine whether or not solid waste situations in rural and urban areas are significantly different. The results revealed that location significantly affects solid waste management in Ghana. Urban communities had lower mean scores than rural communities for poor solid waste situation in homes. However, urban communities had higher mean scores than rural communities for poor solid waste situation in principal streets and dumping sites. The study recommends that the local government authorities implement very comprehensive policies (sanitary inspection, infrastructure development, and community participation) that will take into consideration the specific solid waste management needs of both urban and rural areas.

## 1. Introduction

Sustaining effective solid waste management practices is crucial to both developed and developing countries. Waste management practices, especially the solid waste, differ significantly for developed and developing countries, for urban and rural areas, and for residential, commercial, and industrial producers [1]. For instance, in Ghana, urban domestic waste collection services are often provided by local government authorities or by private companies for a fee while the rural residents dump their solid waste on open dumping sites for free [2, 3]. This is as a result of the general assumption by various governments that the rural people do not have the purchasing power to pay for the solid waste disposal services. However, the repercussions of this act are mostly immeasurable as the open dumping methods create unsightly scenes which degenerate into various poor environmentally related diseases such as malaria, typhoid, and cholera. This makes the current environmental sanitation status of Ghana serious as less than 40% of urban residents are served by a solid waste collection services, less than 30% have acceptable household toilet facilities, and only about 10% of solid wastes generated are properly disposed [4, 5], with rural dwellers less well served [3, 6].

It must be emphasized again that the organisation of solid waste management differs significantly between rural and urban centers in terms of sources, composition, storage, and collection. Various studies have shown major sources of solid waste in urban Ghana in an order of domestic, commercial (including institutions), and industrial, respectively [4, 7–10]. The domestic sources include single family and multiple families and low, medium, and high apartments dwellings. The commercial sources are stores, restaurants, markets, office buildings, hotels, motels, print shops, auto repair shops, medical facilities, and other institutions; while industrial constitutes construction, fabrication, light and heavy manufacturing, refineries, mining, and power plant demolishing [10]. Besides, the domestic remains the highest source of solid waste in the rural areas in Ghana. This is followed by the industrial sources [11].

The composition of solid waste in the urban centers in Ghana is predominantly made of organic (biodegradable) materials and high percentage of plastic waste followed by inert materials which include wood ash, sand, and charcoal [4, 8, 10, 12]. It must be noted that there has been rapid increase in inorganic (plastic waste) waste as a result of changing consumption patterns of the urban dwellers. Again, the organic waste remains the highest in the solid waste composition in the rural areas [1, 2]. This can be attributed to the general household consumption pattern in the rural areas where fresh food items like fruits, tubers, roots, and vegetables form the bulk of purchases of the average household. The processing and consumption of these food items generate a lot of organic waste in the home.

The storage of solid waste before disposal is also crucial in the organisation of solid waste in rural and urban Ghana. The storage of wastes generated by households before collection and transportation to the dump involves the use of various receptacles. These receptacles include polythene bags, propylene sacks, metal bins, and disposing waste into pits dug at the back of the house [10].

According to [11], in the Accra Metropolis, solid waste was stored in polythene bags, cardboard boxes, and old buckets, which was quite prevalent in both the low and middle-income areas and the standard plastic containers were prevalent in the high-income neighbourhoods. Another observation made in the Kumasi Metropolis also showed that though some middle-income residents claimed to be using standard containers, practical observation revealed the use of impoverished galvanized containers, possibly due to the high cost of the former [4, 11]. While generally the urban areas seem to have relatively better storage before disposal, the rural areas do not. They store their solid waste in an open old basket or even put them on the ground before disposal [1, 2].

Means of solid waste collection is another critical component of waste management which results in the passage of waste materials from the source of production to either the point of treatment or final disposal site [13]. Effective means of solid waste collection depends on vehicle type, capacities, and staffing levels. In Ghana, solid waste delivery has evolved through various strategies and methods of collection under different political administrations, from local authority led delivery of services to private sector service delivery. Waste collection became a viable venture from the early 1990s when the German Government supported the Accra Assembly to collect waste from various residential areas and central business district [14, 15]. Although the waste collection rate has improved over the past decade due to greater private sector participation, waste services in low-income areas are still inadequate [11]. Many factors that jointly account for this include institutional weakness, inadequate financing, poor cost recovery, the lack of clearly defined roles of stakeholders, and the lax attitude of officials and residents [16]. Waste collection methods vary widely between these two settlements. There are two main types of waste collection services that are delivered by the authorities in the cities. These are house-to-house and communal collection services [17]. However, there are other methods which include open dumping (traditional method) and road side collection method. While majority of urban dwellers resort to communal containers for their disposal, rural residents resort to open dumping (traditional method) method for their disposal [1, 2]. The privatisation of the solid waste collection activities is actively pursued by local government authorities in the urban cities while the rural areas are offering the traditional free services through the open dumping method.

Literature has copiously advanced arguments for the solid waste management situations in the urban areas and the sources, characterisation, and the challenges associated with their disposal [4, 7–10]. However, in recent times, there has been increasing level of rural consumerism as a result of the role of the rural areas as the market for urban goods hence the comparative consideration of the situation within the rural context. Although the rural areas are the potential periurban fringes that expand to ultimately become the urban centers, research attention in that direction has remained low. With this, it is empirically crucial to analyse the spatial variation between rural and urban solid waste management [16, 18]. The comparative analysis between the rural and urban solid waste scenarios would proffer a holistic policy prescriptions of managing the menace of the solid waste, without overconcentration of efforts in the urban areas compared to the neglect of the rural settings. The aim of the study was to examine the nexus between location and solid waste management practices in Ghana.

## 2. Materials and Methods

**Figure 1:**
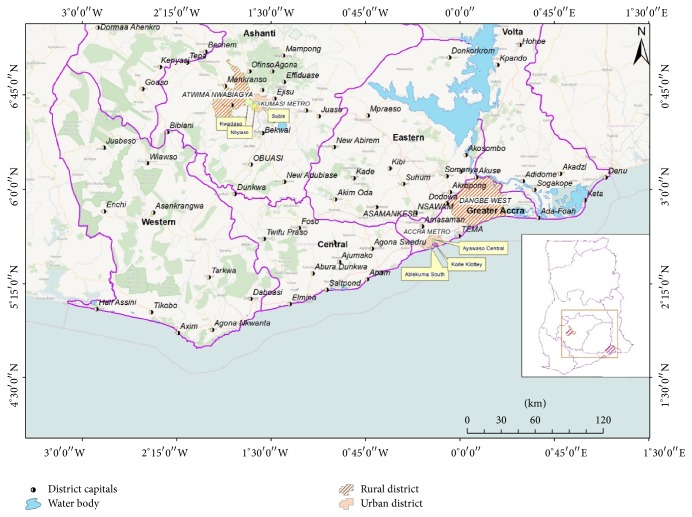
Map showing the study areas. Source: University of Ghana, GIS Laboratory, 2016.

A map showing the study areas is provided in Figure 1.

### 2.1. Sampling Design and Data Collection

Greater Accra and Ashanti Regions are the densely populated regions with cultural-mixed and diverse demographic and socioeconomic indicators in Ghana and therefore appropriate for the study. To reflect the variance in demographic and socioeconomic status, two contrasting districts in each region were chosen, inter alia rural and urban districts. In Ashanti region, Atwima Nwabiagya District and Kumasi Metropolis were purposely selected as rural and urban districts, respectively, while, in Greater Accra Region, Dangbe West District and Accra Metropolis were purposively selected as rural district and urban district, respectively. Kwadaso, Nhyiaeso, and Subin submetros were chosen from Kumasi Metropolis while Ablekuma South, Osu Klottey, and Ayawaso Central submetros were chosen from Accra Metropolis. All communities in the submetros and rural districts that were chosen and the household population are shown in Table 1.

The study adopted [19] as in (1) to calculate the sample size. In all, 400 households were selected from the study areas. The sample size in each district or submetro was on the basis of proportional representation as indicated in Table 2.

(1) $$\begin{array}{c} n=\frac{N}{1+Ne^{2}}, \end{array}$$

where *n* is sample size, *N* is population of households for all communities, and *e* is level of precision (95%).

The study systematically sampled houses from which households and respondents were randomly selected. The sample interval depended on the number of houses. This was done to avoid concentration of respondents in few houses and to ensure fair distribution of sample across districts or submetros. All public structures such as schools and boarding houses, hospital, clinic, maternity homes, restaurants, police and military barracks, and prayer camps were excluded.

Face-to-face interviewer-administered structured questionnaires were used to collect field data. The research instrument was pretested in Tafo submetro, an urban district, and Kwabre East District, a rural district, where 10 houses and 10 households were selected from each. Research assistants from Department of Geography and Rural Development in Kwame Nkrumah University of Science and Technology were trained to assist in the data collection. Researchers officially sought the consent of opinion leaders, especially chiefs in each community, and participants were informed of the purpose of the study and nature and extent of their engagement in the study.

### 2.2. Operationalization and Coding of Study Variables

The study employed cross-sectional survey covering both rural and urban districts in the Ashanti and Greater Accra Regions of Ghana. The study involved adults aged at least 18 years who had lived in their respective communities for at least 5 years. These people had in-depth knowledge about the solid waste situation and management in their respective communities. The dependent variable for the study was solid waste situation while the explanatory variable was location which was coded as 1 for rural community and 2 for urban community. The demographic, socioeconomic, and solid waste situation variables of the study were described as operationalized in Table 2.

### 2.3. Data Analysis

Data were verified and entered into an electronic database and analysed statistically through the SPSS, version 21.0. Descriptive statistics were carried out to describe the background characteristics of the study sample. Pearson's Chi square (*χ* ^2^) test was used to compare demographic and socioeconomic variables in rural and urban areas. Multivariate Test, Tests of Between-Subjects Effects, and Pair-Wise Comparisons were performed through one-way MANOVA to determine whether or not solid waste situations in rural and urban areas are different. MANOVA was appropriate in the paper because solid waste situation (dependent variables) was subgrouped into principal streets, market centers/commercial centers, lorry parks/terminals, dumping sites or landfill sites, and homes, and significance test of each was determined together as a group [20]. Stevens [21] emphasized that with MANOVA relationship among the subgroup dependent variables could be determined and this is what this paper sought to do. The interpretation of the tests results took into consideration the error margin of less than 5% as significant.

## 3. Results and Discussions 

### 3.1. Demography of Respondents

The data reveals no significant difference in sex distribution (*p* = 0.052) and religion (*p* = 0.054) between rural and urban communities. However, the result shows a significant difference in age distribution between rural and urban communities (*p* < 0.001).  Table 3 shows that 86.5 percent of respondents in the rural communities were above 40 years, while 61.3 percent of the respondents in the urban communities were below 40 percent. This suggests more youthful and growing population in urban communities mainly because of migration of youth into the urban communities for perceived white collar jobs. This is because urban communities are the engine of economic growth and provide jobs, services, and the promise of a better life. This increases the quantum of solid waste generation in the urban communities than the rural communities. Educational level (*p* < 0.001), household size (*p* = 0.037), and marital status (*p* < 0.001) were significantly different in urban and rural communities.  Table 3 reveals that 75.6 percent of the respondents in the rural communities had presecondary school education while 79.2 percent of the respondents in urban communities had postsecondary education. The educational levels among the residents can significantly affect the success of solid waste management awareness programmes aimed at improving solid waste management. Information about waste disposal, including labels on waste containers and educational campaign leaflets such as those issued by the Waste Management Department, is usually transmitted in written form and so the ability to read and understand such information is very essential for the effective participation of respondents in the waste management programme of both urban and rural settlements. The current poor solid waste situation in the communities can largely be attributed to the relatively low educational attainment in Ghana. The rural communities significantly had larger household sizes than urban communities. This leads to a high per household waste generation in the rural communities. This may explain why solid waste situation in the rural communities is increasingly emerging as a problem, a situation which was far from existence two decades ago.  Table 3 further indicates that 29.2 percent and 70.8 percent of respondents in the urban communities were single and married, respectively, while 50.5 percent and 49.5 percent in the urban communities were single and married, respectively. Economic hustle in the urban communities makes bread-and-butter issue a priority for many residents, as against a cherished social and cultural (marriage) status in the rural communities. This has a significant implication for solid waste management as marital status affects solid waste management positively [1, 4].

### 3.2. Solid Waste Situation in Urban and Rural Communities

Table 4 shows sources, composition, means of solid waste storage, and solid waste collection arrangements in both rural and urban communities. The commonest source of solid waste was domestic (67.8 percent), followed by commercial (23.5 percent), institutional (4.8 percent), and industrial (4 percent). Domestic source of solid waste was common in both rural (92.4 percent) and urban (46.8 percent) communities; but urban communities had other major source as commercial (37 percent). The sources of solid waste were significantly different between urban and rural communities (*p* < 0.001). The commercial source of solid waste in the urban communities is significant because of the active commercial activities such as trading and restaurants in the urban areas. These findings support the previous studies by [4, 8–10].

Table 4 further indicates a significant difference in composition of solid waste between urban and rural communities (*p* < 0.001). The urban communities had putrescible (50.5 percent), plastic (28.7), papers (12 percent), and inert waste (3.7 percent) as main constituents of solid waste. The rural communities, however, had putrescible (63.6 percent) and plastic (nonputrescible) (36.4 percent) as solid waste components. That notwithstanding, both the urban and rural areas had putrescible waste as the major component of their solid waste composition. This can be attributed to the general household consumption pattern in both urban and rural areas where fresh food items like fruits, tubers, roots, and vegetables form the bulk of purchases of the average household [1, 8, 10]. The processing and consumption of these food items generate a lot of putrescible waste in the home.

Table 4 again indicates that urban communities stored solid waste by use of open containers (9.7), closed containers (80.6), and polythene bags or sacks (9.7). However, rural communities stored solid waste by use of open containers (61.4), closed containers (28.3), and polythene bags or sacks (10.3). The Chi square test result (*p* < 0.001) shows a significant difference in solid waste storage between urban and rural communities. Uncovered waste containers frequently attract animals like dogs, goats, and sheep, as well as rodents which rummage for food in the waste piles which are usually left at the roadside [11, 16]. The result of this is breeding of mosquitoes which causes malaria and other diseases in both urban and rural areas [11, 16].

Solid waste collection arrangement significantly differs in urban and rural communities (*p* < 0.001). Open dumping was the major solid waste collection arrangement in the rural (78.3 percent) communities while communal container collection (37.5 percent) was the major solid waste collection arrangement in the urban communities. This corroborates the findings of [4, 10] that urban communities have the convenience of regular communal container waste collection while rural communities resort to open dumping methods leaving them to their fate, with waste engulfing their communities and homes.

### 3.3. The Locational Response to Solid Waste Situation

The Multivariate Test in Table 5 shows a significant difference in solid waste situation based on location (urban or rural), *F*(5,394) = 40.355, *p* < 0.001; Wilk's Λ = 0.661, partial *η* ^2^ = 0.339. The study reveals that there is a spatial disparity that exists between urban and rural communities with respect to solid waste situation and organisation of solid waste management. While the general solid waste situation in the country is bad, the urban communities receive majority of the investments made on solid waste management at the expense of the rural communities. Meanwhile, the study found that the issue of solid waste management is increasingly becoming a problem in the rural areas of Ghana.

Location had a significant effect on solid waste situation in principal street (*F*(1,398) = 13.490; *p* < 0.001; partial *η* ^2^ = 0.033), market centers (*F*(1,398) = 4.479; *p* = 0.031; partial *η* ^2^ = 0.012), lorry parks (*F*(1,398) = 78.737; *p* < 0.001; partial *η* ^2^ = 0.165), and dumping site (*F*(1,398) = 7.240; *p* = 0.007; partial *η* ^2^ = 0.018) (see Table 6). This confirms an assertion by [16, 18] that principal street and major market centers in urban communities are engulfed with waste compared to rural communities. However, location did not significantly influence solid waste situation in homes (*F*(1,398) = 0.124; *p* = 0.725; partial *η* ^2^ = 0.000). The work in [4] opined that most urban homes are clean in Ghana due to mass personal and home hygiene education.

From Table 7, urban communities (*p* < 0.001) had higher mean scores (*M *= 2.4239) than rural communities (*M *= 2.4239) for poor solid waste situation in principal streets. This implies that the principal streets in urban communities in Ghana are dirtier than that of the rural communities. This is as a result of rapid urbanisation and population growth rate. The rest include institutional flaws, insufficient financing, poor cost recovery, the lack of clearly defined roles of stakeholders, and the lax attitude of residents [11, 16].

The dumping sites in urban communities had more solid waste problems than the rural communities and this was significant (*p* < 0.001). This is as a result of rapid increase in the volume of solid waste generation due to rapid urbanisation and population increase. The changing consumption pattern of urban dwellers has also contributed to the massive generation of plastic (nonputrescible) waste causing a lot of management problems to the urban solid waste managers. However, the urban homes were significantly neater than the rural homes (*p* < 0.001).

## 4. Conclusion and Policy Recommendation 

The principal argument in this paper is that location significantly affects the solid waste situation in Ghana. The study has confirmed that spatial disparities exist in solid waste situation between urban and rural settlements in Ghana. Based on the obtained results from this study, there is a significant dichotomy between the solid waste management organisation in rural and urban areas. The paper has shown that although urban areas get the better services than rural areas with respect to solid waste management, the former still have serious problems to contend with. Results of solid waste composition analysis conducted during the study also revealed that the organic (putrescible) waste was the most prevalent in both urban and rural communities, followed by the nonputrescible waste (plastic). The high proportion of putrescible components in the waste stream can be explained by the fact that there is a high level of consumption of fresh food products from the farm. Furthermore, most of the staple food products yield a lot of waste during preparation and consumption. Again, those households which listed paper and plastic (nonputrescible) as the most frequently generated waste materials engaged in some kind of home-based commercial activities in which paper or plastic was used as raw materials. The paper further identified three major modes of storage for household solid waste in the study areas. These are the use of open container, closed container, and polythene bags. Again, the paper has also shown important findings that have implications for solid waste management. First, the means of collection services that are provided are communal container, open dumping, and roadside collection, respectively. While the communal container collection is the most common in the urban areas, open refuse dumping site is the common practice in the rural areas. Although the private sector is involved in the collection and disposal of the solid waste that is generated, the current situation still leaves much to be desired. This assertion is as a result of tonnes of uncollected solid waste which undoubtedly pose serious environmental hazards. The paper opines that this condition is a recipe for serious outbreak of diseases in urban and rural areas. Generally, the paper reveals that the solid waste situation in the urban areas is poor as compared to the rural areas. Though the urban areas seem to have better services, they still have problems with solid waste management. This may be as a result of rapid urbanisation and changing consumption patterns in the urban areas. It also revealed that solid waste management is increasingly emerging as a problem in the rural areas, a situation which was far from existence two decades ago.

The study recommends a very comprehensive policy approach (sanitary inspection, infrastructure development, and community participation) that will take into consideration the specific solid waste management needs of both urban and rural areas. As solid waste management is increasingly becoming a problem in the rural communities, perhaps as a first step, the local government authorities need to integrate the operations of the hitherto neglected rural solid waste management into the overall solid waste management system. The local government authorities need to give recognition to and incorporate all communities (urban and rural) to make them entitled to waste disposal and other environmental services. This is because public health and the environment cannot be protected without extending basic environmental services to all localities in Ghana. Again, the central government ought to create a national database on waste and also support local government authorities to undertake regular research to generate accurate data on the waste situations within their jurisdictions to facilitate waste planning and management. For instance, accurate data on waste generation and composition would be useful in determining appropriate strategies for waste management.

In addition, a realistic policy framework must be formulated to guide Waste Management Institutions as well as provide them with adequate legal support to enforce their mandates. For example, there is the need to strengthen waste management institution to effectively implement the sanitary inspection policy. Again, the local government authorities must engage the communities especially the rural areas with tact to revive communal labour and self-help activities to ameliorate the numerous solid waste management problems that exist in the urban areas and are increasingly emerging in the rural communities. The local government authorities need to prioritise infrastructure development for solid waste management from their limited resources to enable them to manage solid waste effectively.

## Figures and Tables

**Table 1:** Population of household.

Districts/submetros	Household population	Sample size (proportional rep.)
Ablekuma South	5791369	
Osu Klottey	35508	42
Ayawaso Central	39116	46
Kwadaso	60235	71
Nhyiaeso	34624	41
Subin	48105	57
Atwima Nwabiagya	35205	41
Dangbe West	26489	33

Source: GSS, 2012.

**(a):** 

Variables	Operational definition	Category	Code
Sex (dichotomous)	Being male or female.	Male Female	1 2

Age (ranked)	Number of years of respondents at the time of study.	≤20 20–29 30–39 40–49 50–59 ≥60	1 2 3 4 5 6

Marital status (dichotomous)	Being single or married. Widow/widower/divorced were deemed single while cohabitation was deemed married.	Single Married	1 2

Level of education (nominal)	Completed grade of schooling.	Not attending school Basic education Secondary education Tertiary education	1 2 3 4

Religion (nominal)	Religious affiliation of the respondents. Religions other than Christianity and Islam were classified as others.	Christianity Islam Others	1 2 3

Household size (ranked)	Number of people within household.	≤3 3–5 ≥5	1 2 3

Income level (ranked)	Income of household per month consisting of both cash and kind from all sources within the month.	≤300 301–500 501–800 801–1000 1001–1500 ≥1501	1 2 3 4 5 6

Source: authors' own construct, 2015.

**(b):** 

Variables	Operational definition	Category	Code
Waste situation in Ghana (ranked)	Perception of respondents on the poor state of solid waste in specific areas (principal streets, market centers/commercial centers, lorry parks/terminals, dumping sites or landfill sites, and homes).	Poor Satisfactory Good Very good	1 2 3 4

Sources of solid waste (nominal)	It is defined as most common sources of solid waste being generated in the communities.	Institutional Industrial Commercial Domestic	1 2 3 4

Composition of solid waste (nominal)	Defined as what commonly makes up solid waste generated in the communities.	Putrescible Paper Plastic Metal Inert waste Textile and leather	1 2 3 4 5 6

Means of solid waste storage (nominal)	How solid waste is stored before disposal in the communities.	Open container Closed container Polythene bags or sacks	1 2 3 4

Waste collection arrangement (nominal)	Means through which solid waste is disposed.	Open dump Central container Home collection Roadside collection Others	1 2 3 4 5

Source: authors' own construct, 2015.

**Table 3:** Results of Chi square test for demographic and socioeconomic characteristics and location of respondents.

Variable	Category	Urban community *n* (%)	Rural community *n* (%)	Total *n* (%)	*p* value
Sex	Male Female Total	135 (62.5) 81 (37.5) 216 (100.0)	108 (58.7) 76 (41.3) 184 (100.0)	243 (60.8) 157 (39.2) 400 (100.0)	0.052

Age	≤20 20–29 30–39 40–49 50–59 Total	0 (0.0) 71 (32.1) 63 (29.2) 80 (37.0) 2 (0.9) 216 (100.0)	17 (9.2) 0 (0.0) 8 (4.3) 68 (37.0) 91 (49.5) 184 (100.0)	17 (4.3) 71 (17.8) 71 (17.8) 148 (37.0) 93 (23.3) 400 (100.0)	0.000

Education level	Not attending school Basic education Secondary education Tertiary education Total	3 (1.4) 15 (7.0) 27 (12.5) 171 (79.2) 216 (100.0)	27 (14.7) 112 (60.9) 19 (10.3) 26 (14.1) 184 (100.0)	30 (7.5) 127 (31.8) 46 (11.5) 197 (49.2) 400 (100.0)	0.000

Household size	≤3 3–5 ≥5 Total	83 (38.4) 93 (41.3) 40 (20.3) 216 (100.0)	33 (18.0) 78 (42.4) 73 (39.6) 184 (100.0)	116 (29.0) 171 (42.8) 113 (28.2) 400 (100.0)	0.037

Marital status	Single Married Total	63 (29.2) 153 (70.8) 216 (100.0)	93 (50.5) 91 (49.5) 184 (100.0)	156 (39.0) 244 (61.0) 400 (100.0)	0.000

Religion	Christianity Islamic Others Total	207 (95.8) 9 (4.2) 0 (0.0) 216 (100.0)	104 (56.5) 78 (42.4) 2 (1.1) 184 (100.0)	311 (77.8) 87 (21.8) 2 (0.4) 400 (100.0)	0.054

Source: authors' survey, 2015.

**Table 4:** Chi square test for solid waste situation and location.

Variable	Category	Urban communities	Rural communities	Total	*p* value
Sources	Institutional Industrial Commercial Domestic Total	19 (8.8) 16 (7.4) 80 (37.0) 101 (46.8) 216 (100.0)	0 (0.0) 0 (0.0) 14 (7.6) 170 (92.4) 184 (100.0)	19 (4.8) 16 (4.0) 94 (23.5) 271 (67.8) 400 (100.0)	0.000

Composition	Putrescible Paper Plastic Metal Inert waste Textile and leather Total	109 (50.5) 26 (12.0) 62 (28.7) 11 (5.1) 8 (3.7) 0 (0.0) 216 (100.0)	117 (63.6) 0 (0.0) 67 (36.4) 0 (0.0) 0 (0.0) 0 (0.0) 184 (100.0)	226 (56.5) 26 (6.5) 129 (32.2) 11 (2.8) 8 (2.0) 0 (0.0) 400 (100.0)	0.000

Means of solid waste storage	Open container Closed container Polythene bags or sacks Total	21 (9.7) 174 (80.6) 21 (9.7) 216 (100.0)	113 (61.4) 52 (28.3) 19 (10.3) 184 (100.0)	134 (33.5) 226 (56.5) 40 (10.0)	0.000

Means of waste collection	Open dumping Communal container Home collection Roadside collection Total	61 (28.2) 81 (37.5) 17 (7.9) 57 (26.4) 216 (100.0)	144 (78.3) 40 (21.7) 0 (0.0) 0 (0.0) 184 (100.0)	205 (51.2) 97 (24.2) 17 (4.2) 81 (20.2) 400 (100.0)	0.000

Source: field survey, 2015.

**Table 5:** Multivariate test.

Effect		Value	*F*	Hypothesis Df	Error df	*p* value	Partial eta square
Intercept	Pillai's Trace Wilks' Lambda Hotelling's Trace Roy's Largest Root	0.979 0.021 46.878 46.878	3694.003 3694.003 3694.003 3694.003	5.000 5.000 5.000 5.000	394.000 394.000 394.000 394.000	0.000 0.000 0.000 0.000	0.979 0.979 0.979 0.979

Location	Pillai's Trace Wilks' Lambda Hotelling's Trace Roy's Largest Root	0.339 0.661 0.512 0.512	40.355 40.355 40.355 40.355	5.000 5.000 5.000 5.000	394.000 394.000 394.000 394.000	0.000 0.000 0.000 0.000	0.339 0.339 0.339 0.339

Source: field survey, 2015.

**(a):** 

Source	Dependent variables	Type III sum of square	Df	Mean square	*F*	*p* value	Partial eta square
Corrected model	Principal street	16.039	1	16.039	14.490	0.000	0.033
	Mkt center	2.424	1	2.424	4.679	0.031	0.012
	Lorry parks	30.224	1	30.224	78.737	0.000	0.165
	Dump sites	2.841	1	2.841	7.240	0.007	0.018
	Homes	0.186	1	0.186	0.124	0.725	0.000

Intercept	Principal street	3853.799	1	3853.799	3241.352	0.000	0.891
	Mkt center	3057.784	1	3057.784	5901.576	0.000	0.937
	Lorry parks	810.384	1	810.384	2111.146	0.000	0.841
	Dump sites	4712.001	1	4712.001	12009.370	0.000	0.968
	Homes	2505.446	1	2505.446	1668.198	0.000	0.807

Location	Principal street	16.039	1	16.039	13.490	0.000	0.033
	Mkt center	2.424	1	2.424	4.679	0.031	0.012
	Lorry parks	30.224	1	30.224	78.737	0.000	0.165
	Dump sites	2.841	1	2.841	7.240	0.007	0.018
	Homes	0.186	1	0.186	0.124	0.725	0.000

Error	Principal street	473.201	398	1.189			
	Mkt center	206.216	398	0.518			
	Lorry parks	152.776	398	0.384			
	Dump sites	156.159	398	0.392			
	Homes	597.751	398	1.502			

Source: field survey, 2015.

**(b):** 

Source	Dependent variables	Type III sum of square	Df
Total	Principal street	4408.000	400
	Mkt center	3300.000	400
	Lorry parks	1024.000	400
	Dump sites	4920.000	400
	Homes	3123.000	400

Corrected Total	Principal street	489.240	399
	Mkt center	208.640	399
	Lorry parks	183.000	399
	Dump sites	159.000	399
	Homes	597.937	399

Source: field survey, 2015.

**Table 7:** Pair-Wise Comparisons.

Dependent variable	(I) Location	(J) Location	Mean	Mean difference (I-J)	Std. error	*p* value	95% Conf. interval for difference	
							Lower bound	Upper bounds
Principal street	Urban Rural	Rural Urban	3.7315 2.4239	1.308^*∗*^ −1.308^*∗*^	0.090 0.090	0.000 0.000	1.131 −1.484	1.484 −1.131

Market center	Urban Rural	Rural Urban	2.7546 2.8098	−0.055 0.055	0.073 0.073	0.448 0.448	−0.198 −0.088	0.088 0.198

Lorry park	Urban Rural	Rural Urban	1.4861 1.4076	0.079 −0.079	0.068 0.068	0.248 0.248	−0.055 −0.212	0.212 0.055

Dumping site	Urban Rural	Rural Urban	3.6204 3.2500	0.370^*∗*^ −0.370^*∗*^	0.061 0.061	0.000 0.000	0.251 −.0490	0.490 −0.251

Homes	Urban Rural	Rural Urban	1.7639 3.3913	−1.627^*∗*^ 1.627^*∗*^	0.092 0.092	0.000 0.000	−1.808 1.447	−1.447 1.808

Source: field survey, 2015; ^*∗*^the mean difference is significant at the 0.05 level.
